# Endometrioid adenocarcinoma concurrent with a blue nevus of the endometrium and uterine cervix: A case report

**DOI:** 10.3892/ol.2013.1575

**Published:** 2013-09-12

**Authors:** MITSUAKI ISHIDA, AKIKO KAGOTANI, KEIKO YOSHIDA, MUNEO IWAI, HIDETOSHI OKABE

**Affiliations:** Department of Clinical Laboratory Medicine and Division of Diagnostic Pathology, Shiga University of Medical Science, Tsukinowa-cho, Seta, Otsu, Shiga 520-2192, Japan

**Keywords:** blue nevus, endometrioid adenocarcinoma, endometrium

## Abstract

A blue nevus is a benign melanocytic lesion that is composed of spindle-shaped pigmented melanocytes. Although the uterine cervix is believed to be the most common extracutaneous location of blue nevi, the occurrence of these lesions in the endometrial stroma has been reported, albeit rarely. The present study describes a case of endometrioid adenocarcinoma concurrent with a blue nevus of the endometrium and uterine cervix. A 58-year-old female presented with abnormal vaginal bleeding. A biopsy from the endometrium revealed an endometrioid adenocarcinoma and subsequently, a total hysterectomy was performed. Histopathological study revealed the proliferation of columnar cells that formed irregularly-shaped tubular and cribriform glands. The neoplastic columnar cells had large, round to oval nuclei containing a single small nucleolus. Focal squamous differentiation was noted. In the stroma of the non-neoplastic endometrium, single or small aggregates of short spindle-shaped cells containing melanin without atypia were observed. These melanocytes were also present in the endocervix. Therefore, the final diagnosis was of endometrioid adenocarcinoma concurrent with a blue nevus of the endometrium and cervix. This is the first documented case of a blue nevus of the endometrium and endocervix. The pathogenesis of blue nevi of the genital tract is not yet completely understood. Possible origins of these cells include Schwann cells or perineural cells of the peripheral nerve fiber or the abnormal migration of neural crest-derived cells.

## Introduction

A blue nevus is a distinct type of benign melanocytic lesion composed of spindle-shaped pigmented melanocytes, which occasionally occurs at extracutaneous sites, including the vagina, uterine cervix, prostate and oral mucosa (1–3). Although the uterine cervix is believed to be the most common extracutaneous location of blue nevi, the occurrence of the disease in the endometrial stroma has been reported, albeit rarely (4,5). The present study describes a case of endometrioid adenocarcinoma concurrent with a blue nevus of the endometrium and uterine cervix. Written informed consent was obtained from the patient.

## Case report

**Figure 1:**
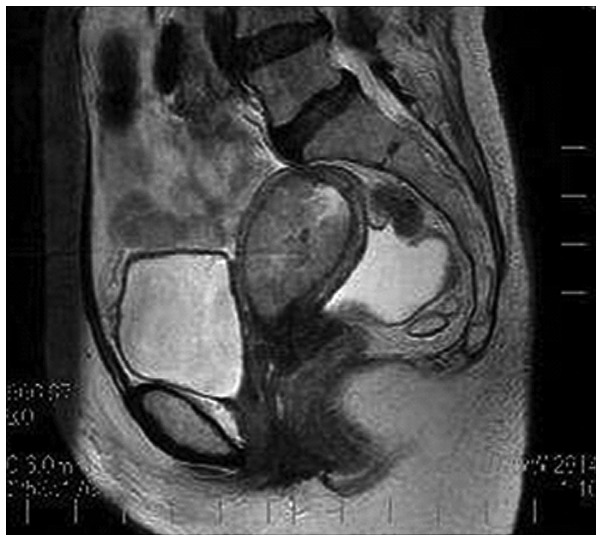
Magnetic resonance imaging showing thickening of the endometrium and a tumorous lesion with enhancement in the uterine corpus wall.

A 58-year-old Japanese female (gravida 2, para 2) presented with abnormal vaginal bleeding. Computed tomography and magnetic resonance imaging demonstrated thickening of the endometrium and a tumorous lesion with enhancement in the uterine corpus wall (Fig. 1). A biopsy of the endometrium revealed an endometrioid adenocarcinoma and subsequently, a total hysterectomy and bilateral salpingo-oophorectomy were performed with dissection of the pelvic lymph nodes.

The post-operative course of the patient was uneventful. No recurrence or metastasis was observed during 14 months of medical follow-up.

The formalin-fixed, paraffin-embedded tissue blocks of the specimens were cut into 3-μm thick sections, deparaffinized and rehydrated. Each section was stained with hematoxylin and eosin. The endometrial and uterine cervical specimens were also stained using Prussian-blue and Fontana-Masson methods. Immunohistochemical analyses were performed using an autostainer (BenchMark XT system; Ventana Medical Systems, Inc., Tucson, AZ, USA) with bleaching pretreatment. The primary antibodies that were used were a mouse monoclonal antibody against CD68 (KP1), a mouse monoclonal antibody against CD163 (10D6) (both Novocastra Laboratories, Ltd., Newcastle upon Tyne, UK), a mouse monoclonal antibody against Melan-A (A103; DakoCytomation, Glostrup, Denmark) and a rabbit polyclonal antibody against S-100 protein (Nichirei, Tokyo, Japan).

**Figure 2:**
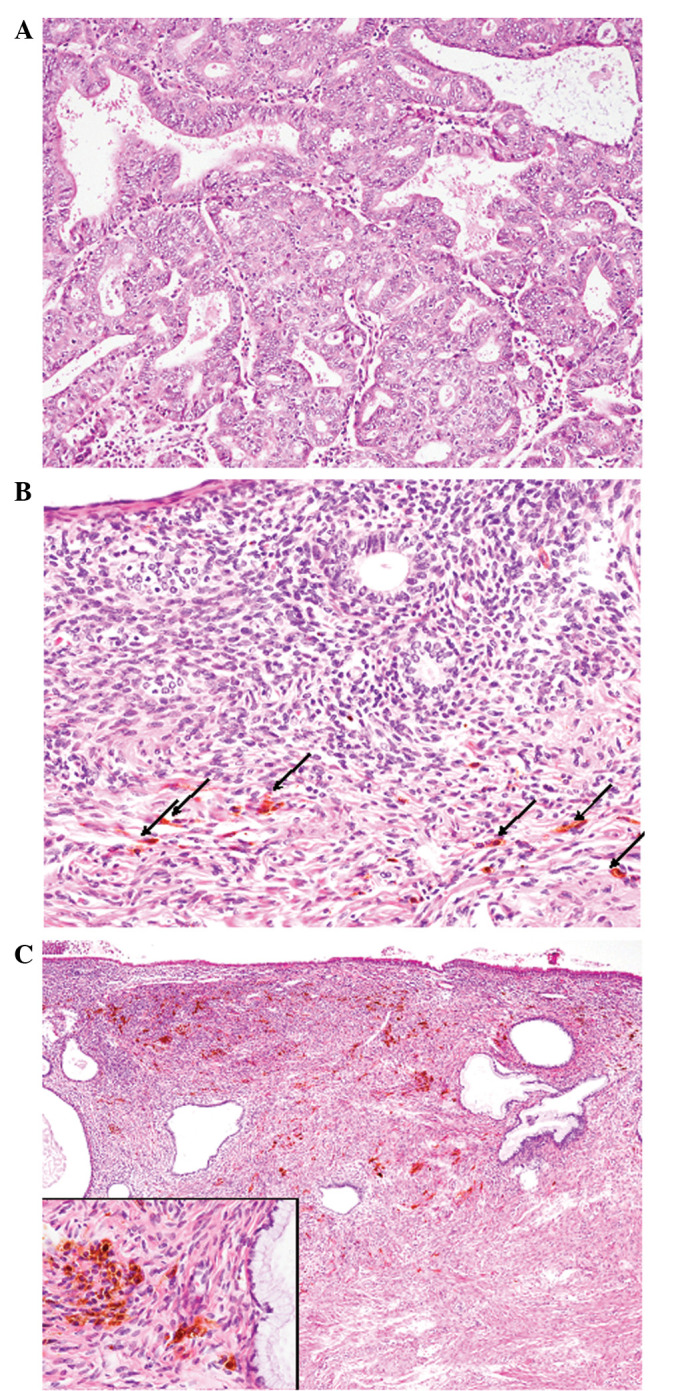
(A) Endometrial tumor. Proliferation of atypical columnar cells containing large round-to-oval nuclei that formed irregularly-shaped tubular and fused/cribriform glands [hematoxylin and eosin (HE) staining; magnification, ×100]. (B) Short spindle-shaped melanocytes in the endometrial stroma (arrows; HE staining; magnification, ×100). (C) Short spindle-shaped or polygonal melanocytes are also present in the stroma of the endocervix. These melanocytes are without atypia (inset) [HE staining; magnification, ×40, (inset) ×200].

Microscopic examination of the resected uterine corpus specimens revealed the proliferation of columnar cells that formed irregularly-shaped tubular and fused/cribriform glands (Fig. 2A). The neoplastic columnar cells had a slightly eosinophilic cytoplasm and large round-to-oval nuclei containing a single small nucleolus (Fig. 2A). No mucin was observed in the cytoplasm of the tumor cells. Mitotic figures were easily observed (25/10 high-power fields). Focal squamous differentiation was noted. However, no solid component was present. The tumor had invaded into less than half of the myometrium and no invasion into the cervix or parametrium was observed. In addition, no lymph node and ovarian metastasis was observed. These histopathological features were typical of an endometrioid adenocarcinoma with squamous differentiation [International Federation of Gynecology and Obstetrics (FIGO) grade 1; pTIb, N0, M0].

In the stroma of the non-neoplastic endometrium, single or small aggregates of short spindle-shaped cells containing granular dark brown pigments were observed (Fig. 2B). These cells were without atypia and contained small round nuclei without nucleoli (Fig. 2B). No mitotic figures were noted. Neither a stromal reaction nor inflammatory cell infiltration was present. The pigment was negative for Prussian-blue stain, but was stained black by the Fontana-Masson method. Therefore, the pigment was considered to be melanin rather than hemosiderin. Furthermore, these melanin-laden cells were immunohistochemically positive for S-100 protein and Melan-A, but negative for CD68 and CD163.

In addition, abundant short spindle-shaped or polygonal cells containing melanin were also observed in the stroma of the uterine cervix (Fig. 2C). According to these findings, the patient was diagnosed with endometrioid adenocarcinoma concurrent with a blue nevus of the endometrium and uterine cervix.

However, melanin was absent in the normal endometrial and endocervical glands, as well as the neoplastic endometrial glands.

## Discussion

The present study describes the first case of endometrioid adenocarcinoma concurrent with a blue nevus of the endometrium and uterine cervix. Melanocytes are usually not present in the normal endometrium and uterine cervix (6). Babes (4) reported the first documented case of melanocytes in the non-neoplastic endometrial stroma within an endometrial polyp in 1927, and Shintaku *et al*(5) reported the second documented case of a blue nevus of the endometrium in a 36-year-old female with long-standing idiopathic amenorrhea or oligomenorrhea. The present study is the first to demonstrate a case of melanin-laden cells identified in the endometrial stroma and uterine cervix.

Blue nevi of the endocervix are also referred to as the ‘foci of stromal melanocytes’ (6), although the former term is more widely used to describe this type of lesion. However, Uehara *et al*(6) proposed that ‘blue nevi’ of the endocervix should be called ‘foci of stromal melanocytes’ of the endocervix, as these lesions consist of scattered irregularly-shaped pigmented spots that are distributed among the cervical glands without nodular formation or exophytic growth, which are analogous to those in dermal melanocytosis, including Mongolian spots or nevi of Ito, rather than cutaneous blue nevi, which usually form solid nodular lesions.

The pathogenesis of blue nevi of the genital tract is not yet completely understood, and furthermore, the pathogenesis of the melanocytic colonization of non-melanocytic lesions, including pigmented cervical intraepithelial neoplasia, also remains unclear (7). However, certain hypotheses have been proposed. One possibility is that these melanocytes may be derived from Schwann cells or perineural cells of the peripheral nerve fibers that are present in the endometrium or endocervix, which have the capacity to synthesize melanin (6). The other possibility is that they may be a result of the abnormal migration of neural crest-derived cells during fetal development (8). Furthermore, Shintaku *et al*(5) speculated that the melanin-laden cells in the endometrial stroma are not true melanocytes, but are altered or transformed endometrial stromal cells, since these cells had cytological features that were indistinguishable from those of the surrounding stromal cells. In the present case, the melanin-laden cells were observed in the endometrium and endocervix. Therefore, these cells were not considered to be altered endometrial stromal cells and were likely to be derived from peripheral nerve fiber cells or the abnormal migration of the neural crest.

In conclusion, the present case is unique, since the endometrioid adenocarcinoma was concurrent with a blue nevus of the endometrium and cervix. Only a limited number of carcinosarcoma cases of the uterine corpus containing melanin-containing cells have been reported (9). The most plausible explanation of this phenomenon in carcinosarcoma is that the neoplastic cells of Müllerian derivation had undergone aberrant neuroectodermal differentiation, resulting in the production of melanin (9). However, in the present case, the occurrence of the endometrioid adenocarcinoma and blue nevus component is believed to be incidental.
